# The Zur regulon of *Corynebacterium glutamicum *ATCC 13032

**DOI:** 10.1186/1471-2164-11-12

**Published:** 2010-01-07

**Authors:** Jasmin Schröder, Nina Jochmann, Dmitry A Rodionov, Andreas Tauch

**Affiliations:** 1 Institut für Genomforschung und Systembiologie, Centrum für Biotechnologie, Universität Bielefeld, D-33615 Bielefeld, Germany; 2 International NRW Graduate School in Bioinformatics and Genome Research, Centrum für Biotechnologie, Universität Bielefeld, D-33615 Bielefeld, Germany; 3 Burnham Institute for Medical Research, La Jolla, CA 92037, USA; 4 Institute for Information Transmission Problems (the Kharkevich Institute), RAS, 127994 Moscow, Russia

## Abstract

**Background:**

Zinc is considered as an essential element for all living organisms, but it can be toxic at large concentrations. Bacteria therefore tightly regulate zinc metabolism. The Cg2502 protein of *Corynebacterium glutamicum *was a candidate to control zinc metabolism in this species, since it was classified as metalloregulator of the zinc uptake regulator (Zur) subgroup of the ferric uptake regulator (Fur) family of DNA-binding transcription regulators.

**Results:**

The *cg2502 *(*zur*) gene was deleted in the chromosome of *C. glutamicum *ATCC 13032 by an allelic exchange procedure to generate the *zur*-deficient mutant *C. glutamicum *JS2502. Whole-genome DNA microarray hybridizations and real-time RT-PCR assays comparing the gene expression in *C. glutamicum *JS2502 with that of the wild-type strain detected 18 genes with enhanced expression in the *zur *mutant. The expression data were combined with results from cross-genome comparisons of shared regulatory sites, revealing the presence of candidate Zur-binding sites in the mapped promoter regions of five transcription units encoding components of potential zinc ABC-type transporters (*cg0041*-*cg0042*/*cg0043*; *cg2911*-*cg2912*-*cg2913*), a putative secreted protein (*cg0040*), a putative oxidoreductase (*cg0795*), and a putative P-loop GTPase of the COG0523 protein family (*cg0794*). Enhanced transcript levels of the respective genes in *C. glutamicum *JS2502 were verified by real-time RT-PCR, and complementation of the mutant with a wild-type *zur *gene reversed the effect of differential gene expression. The zinc-dependent expression of the putative *cg0042 *and *cg2911 *operons was detected *in vivo *with a *gfp *reporter system. Moreover, the zinc-dependent binding of purified Zur protein to double-stranded 40-mer oligonucleotides containing candidate Zur-binding sites was demonstrated *in vitro *by DNA band shift assays.

**Conclusion:**

Whole-genome expression profiling and DNA band shift assays demonstrated that Zur directly represses in a zinc-dependent manner the expression of nine genes organized in five transcription units. Accordingly, the Zur (Cg2502) protein is the key transcription regulator for genes involved in zinc homeostasis in *C. glutamicum*.

## Background

*Corynebacterium glutamicum *is a gram-positive soil bacterium that is well-established for the industrial production of several L-amino acids [1,2]. The complete genome sequence of the type strain *C. glutamicum *ATCC 13032 is available [3], and it was screened by bioinformatic tools to predict the repertoire of DNA-binding transcription regulators in this organism [4,5]. Transcription regulators represent key components in the control of bacterial gene expression and permit the cell to sense and respond to environmental changes [6]. Amongst others, metal ion homeostasis in bacterial cells is tightly regulated by specific metal-sensing transcription regulators. These metalloregulatory proteins, in principle, sense the intracellular levels of specific metal ions by binding them to a metal binding site, which leads to conformational changes affecting the regulator's ability to bind operator sites in regulatory DNA regions [7]. Prominent protein families of metalloregulators are DtxR [8], MerR [9], SmtB/ArsR [10], and Fur [11]. The ferric uptake regulator Fur was originally described as iron-sensing repressor of genes involved in siderophore biosynthesis and iron transport in *Escherichia coli *[12,13], but Fur also activates the expression of many genes by either direct or indirect mechanisms and can be regarded as global transcription regulator of iron homeostasis in *E. coli *[14]. Numerous studies indicated a tremendous diversity in metal selectivity and biological function within the Fur protein family that can be divided into sensors of iron (Fur), manganese (Mur), nickel (Nur), and zinc (Zur) [14].

Zinc is considered an essential nutrient for all living organisms. As zinc can be toxic at large concentrations [15], zinc uptake, efflux, storage, and metabolism is in general tightly regulated in bacteria [16]. During our work on reconstructing the transcriptional regulatory network of *C. glutamicum *[5,17], we recognized the Cg2502 protein as candidate to control the zinc metabolism in this species, since it was classified as DNA-binding transcription regulator of the Fur family [4] and iron metabolism is under global control of the dual regulator Cg2103, a member of the DtxR protein family [18]. In this study, comparative whole-genome DNA microarray hybridizations revealed a set of differentially expressed genes that are under transcriptional control by Cg2502 (now named Zur). Comparative genomic analysis of Zur regulons in actinobacteria detected candidate Zur-binding sites within the mapped promoter regions of potential target genes in *C. glutamicum *ATCC 13032. The DNA binding of Zur to these operator sites occurred in a zinc-dependent manner and was verified by DNA band shift assays, providing clear evidence that Zur is involved in zinc-dependent transcriptional regulation of gene expression in *C. glutamicum *ATCC 13032.

## Results

### Annotation of the corynebacterial zinc uptake regulator Zur

**Figure 1:**
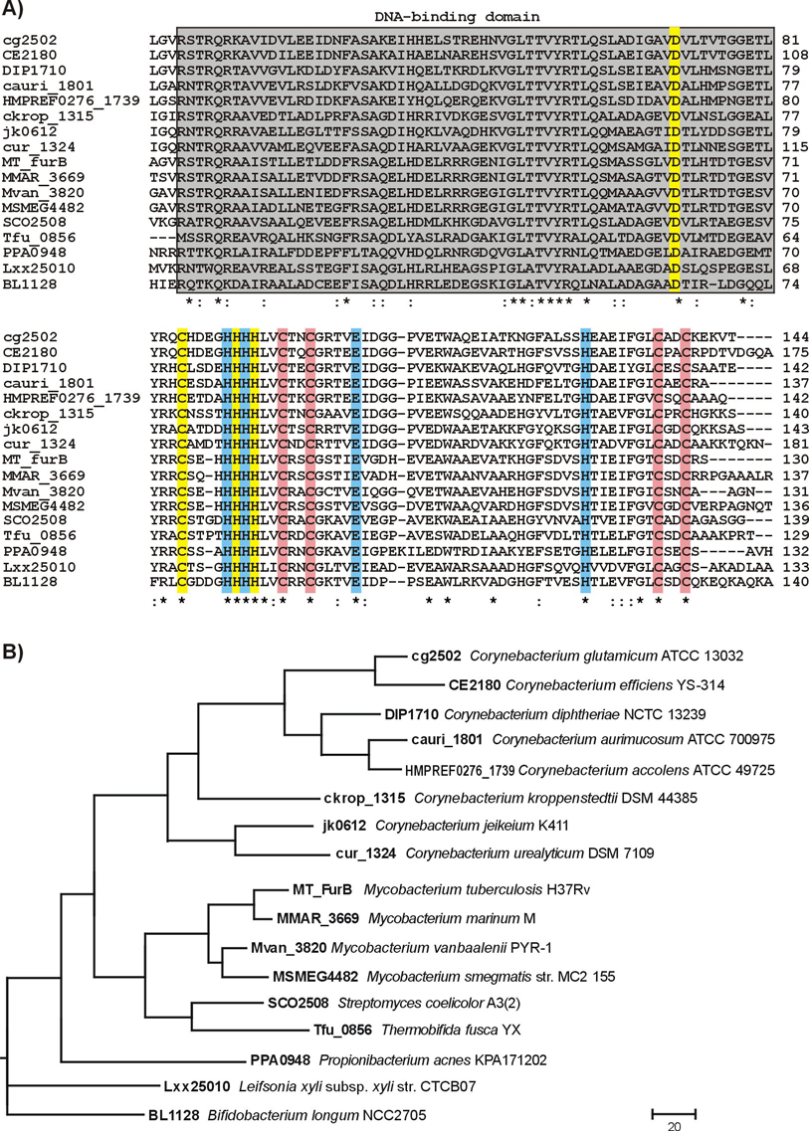
**Comparative analysis of Zur proteins from actinobacteria**. **(A)**, Multiple amino acid sequence alignment of actinobacterial Zur proteins, including FurB from *M. tuberculosis *H37Rv. The winged-helix DNA binding domain is highlighted in grey. Three zinc binding sites (Zn 1 to Zn 3) deduced from the crystal structure of the mycobacterial FurB protein [23] are specifically coloured. Zn 1 (yellow): Asp-71, Cys-85, His-91, and His-93; Zn 2 (red): Cys-96, Cys-99, Cys-136, and Cys-139; Zn 3 (blue): His-90, His-92, Glu-111, and His-128 (according to the *C. glutamicum *protein positions). **(B)**, Maximum likelihood phylogenetic tree of Zur protein orthologues from actinobacteria. The source of the abbreviated Zur-like proteins is indicated by the respective GenBank identifiers.

The Cg2502 (Zur) protein of *C. glutamicum *ATCC 13032 has a predicted size of 144 amino acids, a theoretical molecular mass of 15.7 kDa and belongs to the small core set of 24 transcription regulators that were detected in all hitherto sequenced corynebacterial genomes [5,19]. Protein domain predictions performed with the SUPERFAMILY [20] and the Conserved Domain Database tools [21] showed that the Zur protein contains an amino-terminal helix-turn-helix motif of the winged-helix type and is a member of the Zur (zinc uptake regulator) subgroup of the Fur (ferric uptake regulator) family of metalloregulatory proteins [14]. According to BLASTP data [22], the *C. glutamicum *Zur protein revealed high amino acid sequence similarities to orthologous proteins encoded in other sequenced corynebacterial genomes, ranging from 56% to 80% identical amino acids (Fig. 1A). Furthermore, Zur orthologues in other actinobacteria are well conserved and corroborated by the phylogenetic tree for these proteins (Fig. 1B). The Zur orthologue in *Mycobacterium tuberculosis *H37Rv (57% identity with Cg2502) is the zinc metalloregulator FurB, whose crystal structure has been elucidated recently [23]. The multiple alignment of Zur proteins from actinobacteria demonstrates the conservation of all amino acid residues forming three distinct zinc binding sites in the FurB protein (Fig. 1A). The zinc binding site 1 is surrounded by conserved aspartate, cysteine and histidine residues, whereas the zinc binding site 2 is represented by a cluster of four cysteines. The putative zinc binding site 3 is build by three histidines and one glutamate, but the exact biological function of this protein site remains to be determined [23]. These structural protein data strongly suggested that the Cg2502 (Zur) protein of *C. glutamicum *is a zinc-binding protein and involved in the transcriptional regulation of zinc metabolism in this species.

**Figure 2:**
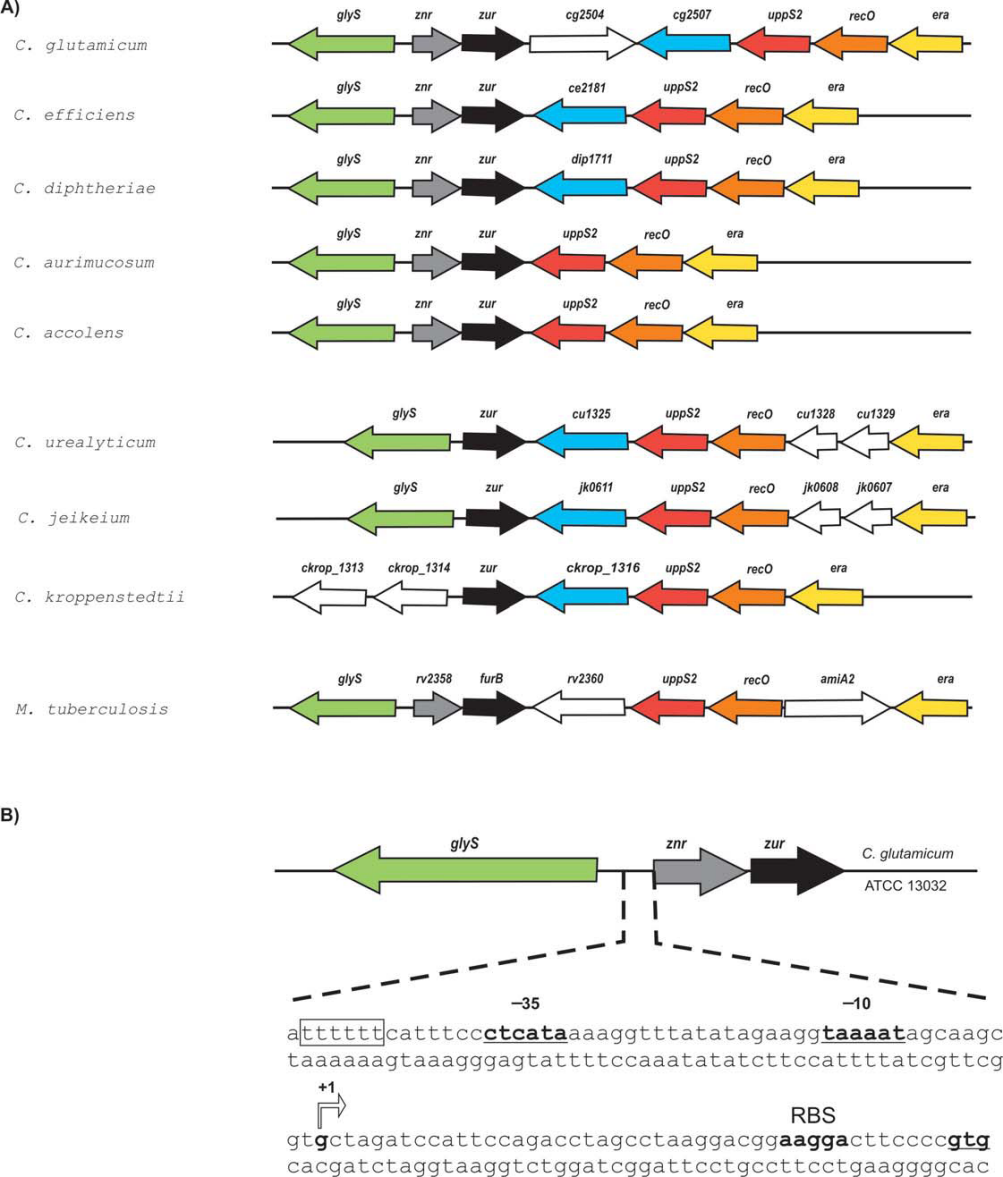
**Genomic organization of the *znr*-*zur *gene region in corynebacterial genomes and *M. tuberculosis *H37Rv**. **(A)**, Comparison of the *znr*-*zur *genome region. The respective gene regions were obtained from *C. glutamicum *ATCC 13032 (NC_006958), *C. efficiens *YS-314 (NC_004369), *C. diphtheriae *NCTC 13239 (NC_002935), *C. aurimucosum *DSM44827 (NC_012590), *C. accolens *ATCC 49725 (NZ_ACGD00000000), *C. urealyticum *DSM7109 (NC_010545), *C. jeikeium *K411 (NC_007164), *C. kroppenstedtii *DSM44385 (NC_012704), and *M. tuberculosis *H37Rv (NC_000962). Orthologous genes are specifically labeled. Please note that the gene regions of *C. jeikeium *and *C. accolens *are shown in reversed orientation. **(B)**, The *znr *upstream region of *C. glutamicum *ATCC 13032. The mapped transcription start site (+1) and the deduced core promoter regions (- 35 and - 10) are marked in bold. A stretch of six thymine residues representing a potential up-element is boxed. A putative ribosome-binding site (RBS) is indicated, the GTG start codon of *znr *is underlined.

According to comparative genomic analysis, the *cg2502 *(*zur*) gene of *C. glutamicum *ATCC 13032 is located in a conserved gene region in all hitherto sequenced corynebacterial genomes (Fig. 2A). In the genomes of *C. glutamicum*, *C. efficiens*, *C. diphtheriae*, *C. aurimucosum*, and *C. accolens*, all representing members of the main lineage of the genus *Corynebacterium *[24,25], the *zur *gene is located downstream of another regulatory gene (*znr*) encoding a putative metal-sensing transcription regulator of the SmtB/ArsR protein family [4,10]. In genomes of corynebacteria belonging to the *C. jeikeium *and *C. kroppenstedtii *branches, the overall location of the *zur *gene is also conserved, but an orthologue of *znr *is lacking in front of the *zur *coding region (Fig. 2A). Since the orthologous protein of Znr from *M. tuberculosis *H37Rv (Rv2358) is apparently involved in zinc-dependent transcriptional (auto)regulation of the *rv2358*-*furB *operon [26], the homologous *znr*-*zur *gene region of *C. glutamicum *ATCC 13032 may also encode the regulatory switches involved in controlling the zinc homeostasis in this organism.

### Transcriptional organization of the *znr-zur* gene region in *C. glutamicum*

**Figure 3:**
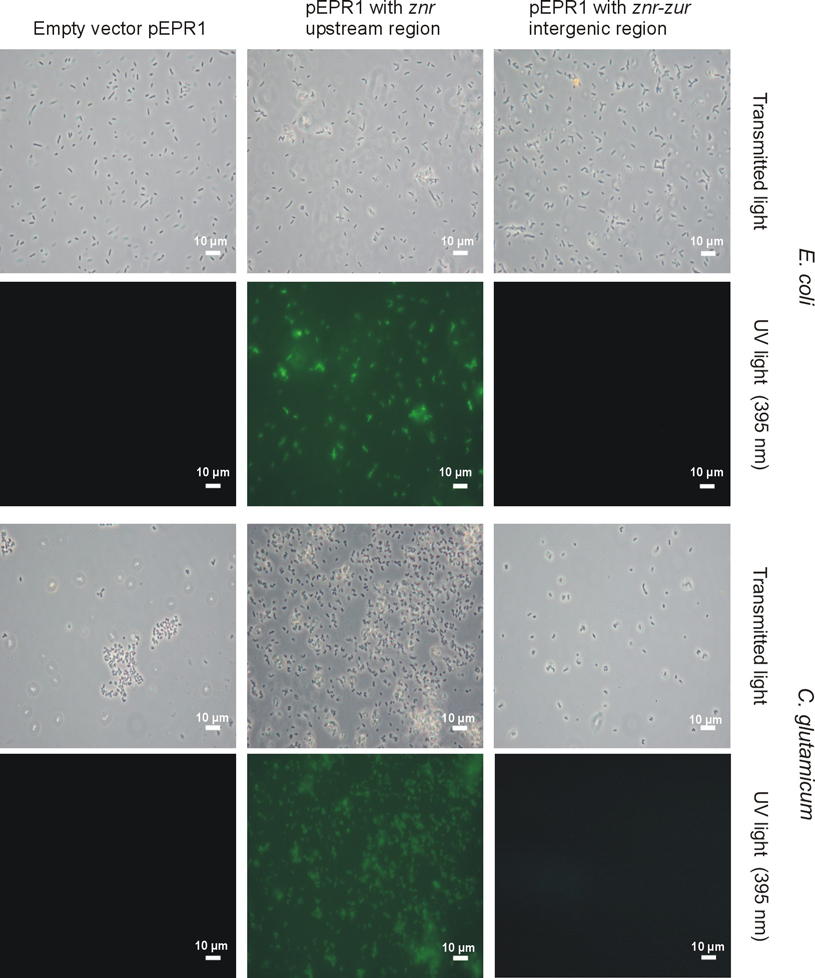
**Fluorescence microscopy of *E. coli *DH5α MCR and *C. glutamicum *ATCC 13032**. The cells are carrying either the empty pEPR1 vector, pEPR1 containing the *znr *upstream region, or pEPR1 containing the *znr*-*zur *intergenic region. Images at a 400-fold magnification were taken with transmitted light or UV light at 395 nm to detect GFP fluorescence.

Operon predictions for *C. glutamicum *ATCC 13032 suggested that the *znr*-*zur *genes are expressed as bicistronic transcript [27]. To provide experimental support for this prediction, the transcription of the *znr*-*zur *region was analyzed by marker gene expression using the green fluorescent protein encoded on the promoter-probe vector pEPR1 [28]. Both the *znr *upstream region and the *znr*-*zur *intergenic region were tested for promoter activity in *E. coli *DH5α MCR and *C. glutamicum *ATCC 13032 (Fig. 3). For this purpose, a 141-bp DNA fragment covering the *znr *upstream region and a 107-bp DNA fragment containing the 40-bp *znr*-*zur *intergenic region were amplified by PCR and cloned in front of the promoterless *gfp *gene present on pEPR1. The expression of *gfp *was detected by fluorescence microscopy only with a pEPR1 derivative containing the *znr *upstream region, indicating the presence of a promoter in front of *znr *and supporting the view that *znr *and *zur *are organized as operon. This observation was further strengthened by detecting with RT-PCR a 309-bp cDNA fragment that encompasses the intergenic region on the *znr*-*zur *transcript (data not shown). The promoter in front of the *znr *gene was deduced from RACE-PCR experiments with total RNA purified from *C. glutamicum *ATCC 13032 cultures, showing that transcription starts at a guanine residue located 45 nucleotides upstream of the GTG start codon of *znr *(Fig. 2B). Based on the known consensus motif for corynebacterial promoters [29], potential -10 (TAAAAT) and -35 (CTCATA) promoter regions with an 18-bp spacing and a putative up-element [30] were detected (Fig. 2B).

### Computational identification of actinobacterial Zur regulons

**Figure 4:**
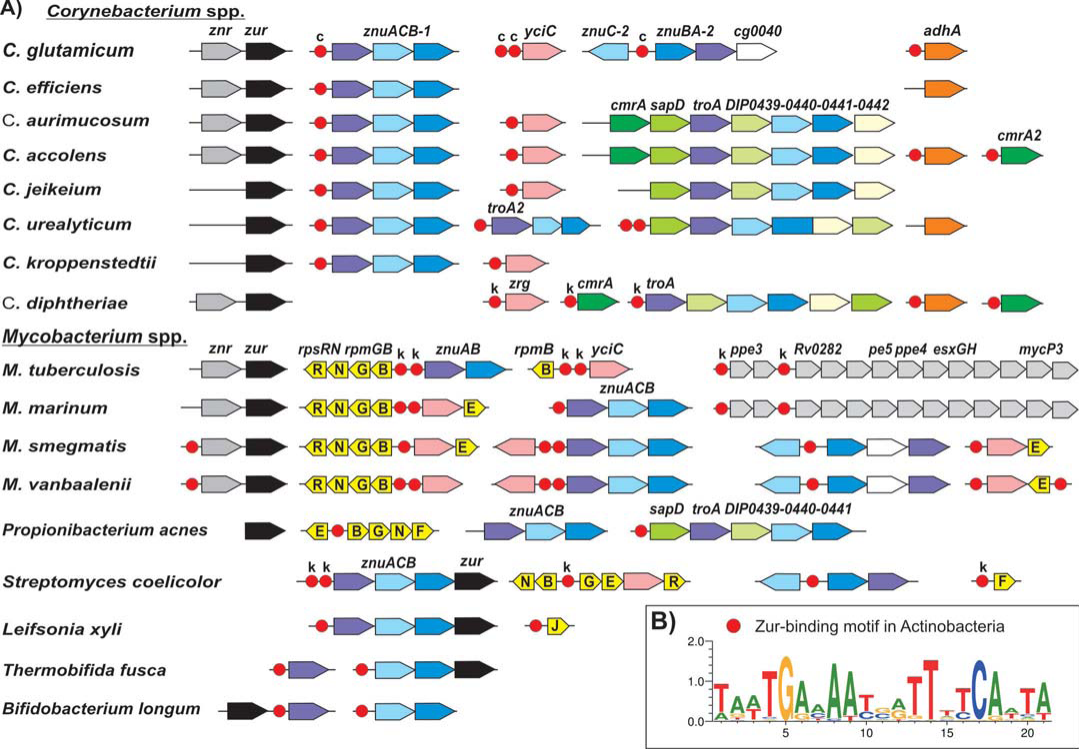
**Predicted actinobacterial Zur regulons**. **(A)**, Chromosomal clusters of predicted Zur-regulated genes and their orthologues in members of the class *Actinobacteria*. The locations of candidate Zur-binding sites are shown by red circles. The Zur-binding sites confirmed in this study are marked with 'c'; the previously known Zur-binding sites are marked with 'k'. Homologous genes are marked by matching colour, including zinc ABC-type transporter *znuABC *(shades of blue), *yciC *for zinc allocation protein (pink), *cmrA *and *sapD *genes for surface-anchored proteins (shades of green), alcohol dehydrogenase *adhA *(orange), genes encoding ribosomal proteins (yellow), *zur *(black), *znr *(dark grey). **(B)**, Consensus sequence logo for the predicted Zur-binding sites.

We applied comparative genomic techniques such as cross-genome comparison of shared regulatory sites [6] to reconstruct Zur regulons in the genomes of eight *Corynebacterium *species, as well as other representative members of the taxonomic class *Actinobacteria *(four *Mycobacterium *species, *Propionibacterium acnes*, *Streptomyces coelicolor*, *Leifsonia xyli*, *Thermobifida fusca*, and *Bifidobacterium longum)*. Initially, we collected the upstream regions of candidate zinc uptake genes (*znuACB*) in the analyzed actinobacterial genomes and applied the motif recognition program SignalX. The identified 21-bp palindromic motif (Fig. 4B) was similar to previously identified Zur-binding motifs in *M. tuberculosis *and *S. coelicolor *[31-33]. We constructed a positional-weight matrix for the identified Zur-binding motif and applied it to scan the genomes of actinobacteria for additional candidate Zur-binding sites. After filtering of false-positive sites by the consistency check approach and accounting for possible operon structures, we combined the final list of predicted members of the Zur regulons in the analyzed genomes of actinobacteria (see additional file 1) (Fig. 4A).

Overall, a conserved core of the reconstructed Zur regulons includes one or multiple paralogues of the zinc ABC-type transporter ZnuACB and a putative P-loop GTPase of the COG0523 family [34], orthologues of the *B. subtilis *YciC protein [35]. In *Mycobacterium *species, *P. acnes*, *S. coelicolor*, and *L. xyli*, the Zur regulon includes paralogues of various ribosomal proteins (RpmB, RpmG, RpmE, RpmF, RpmJ, RpsN, RpsR). These observations are in agreement with the previously described Zur-dependent regulation of ribosomal protein genes in *M. tuberculosis *and *S. coelicolor *[31-33]. The *znr-zur *operon is preceded by a candidate Zur-binding site only in two *Mycobacterium *species (Fig. 4A). The *C. diphtheriae *Zur regulon includes the candidate ABC-type metal transporter operon *troA-sapD-DIP0439-DIP0440-DIP0441-DIP0442 *and the *cmrA *gene encoding a surface-associated protein [36]. Additional candidate Zur-binding sites were detected upstream of the *adhA *gene encoding zinc-dependent alcohol dehydrogenase in *C. glutamicum *[37] and *adhA *orthologues in *C. accolens *and *C. diphtheriae *(Fig. 4A).

### Global gene expression profiling of the* zur*-mutant *C. glutamicum* JS2502

**Figure 5:**
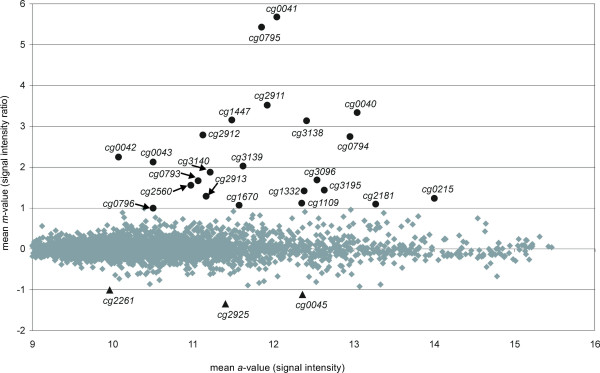
**Ratio/intensity (*m*/*a*) plot deduced from DNA microarray hybridizations comparing the transcriptome of the *zur *mutant *C. glutamicum *JS2502 with that of the wild-type strain *C. glutamicum *ATCC 13032**. Two biological replicates including label swapping were used for DNA microarray hybridizations. Genes showing significantly enhanced expression in *C. glutamicum *JS2502 are marked by black dots, decreased transcript levels are indicated by triangles, and genes without differential expression pattern are shown by grey diamonds. Genes were regarded as differentially expressed using the following cut-offs: *m*-value ≥ 1.0, upregulation; *m*-value ≤- 1.0, downregulation. The cut-offs correspond to relative changes in gene expression of at least two-fold.

To identify *C. glutamicum *genes that are under transcriptional control by Zur, the *zur *gene was deleted in the chromosome of the wild-type strain *C. glutamicum *ATCC 13032 by an allelic exchange procedure, resulting in the mutant strain *C. glutamicum *JS2502. Growth of the *zur*-deficient mutant *C. glutamicum *JS2502 in minimal medium CGXII was indistinguishable from the parental wild-type strain (data not shown), indicating that deregulation of the Zur regulon is not detrimental to any basic physiological functions in *C. glutamicum*. The genome-wide expression profile of *C. glutamicum *JS2502 was compared with that of *C. glutamicum *ATCC 13032 by DNA microarray hybridizations. The resulting ratio/intensity (*m*/*a*) plot of the normalized data, based on two hybridization experiments with label swapping, is presented in Fig. 5. By applying a ratio cut-off of ± 1, which is equivalent to relative expression changes of at least two-fold, 23 genes exhibited higher transcript levels in the *zur *mutant when compared to the wild-type strain, whereas three genes showed lower transcript levels in *C. glutamicum *JS2502.

Among the genes that are up-regulated in the *zur *mutant, we detected five *C. glutamicum *transcription units that are preceded by candidate Zur-binding sites: *cg2911*-*cg2912-cg2913*, *cg0040-cg0041-cg0042*/*cg0043 *and *cg0794/cg0795 *(Table 1). The first two operons encode components of the putative zinc/manganese ABC-type transporter ZnuACB and the putative secreted protein Cg0040, whereas the latter genes encode a P-loop GTPase of the COG0523 protein family (Cg0794 or YciC) and a putative oxidoreductase of unknown physiological function (Cg0795), respectively. The *adhA *gene, encoding a zinc-dependent alcohol dehydrogenase [37] and predicted to be a candidate member of the Zur regulon with a conserved candidate Zur-binding site (AATTGAAAAACATTTCCATTA), was not detected as differentially expressed by DNA microarray hybridizations. In summary, genome-wide expression profiling and motif searches revealed six transcription units of *C. glutamicum *ATCC 13032 that were considered as potential targets for a direct transcriptional control by the zinc-sensing repressor Zur (Table 1).

**Table 1:** Differentially regulated Zur target genes preceded by candidate Zur-binding sites in *C. glutamicum *ATCC 13032.

CDS	Gene	Predicted function	21-bp motif^1^	Differential gene expression^2^	
				Array	RT-PCR
*cg0040*	-	secreted protein	-	3.34	4.5
*cg0041*	*znuA2*	ABC-type Zn/Mn transporter, substrate-binding protein	-	5.68	27900
*cg0042*^3^	*znuB2*	ABC-type Zn/Mn transporter, permease subunit	TAATGATAACGGTTATCATTT	2.25	331
*cg0043*	*znuC2*	ABC-type Zn/Mn transporter, ATPase subunit	AAATGATAACCGTTATCATTA	2.13	50.2
*cg0794*	*yciC*	P-loop GTPase of the COG0523 family	TATTGAAAATGATTCCCAAAA	2.75	10.5
*cg0795*	-	oxidoreductase	TAATGGAAATTGTTTTCAATA	5.43	45500
*cg2911*^4^	*znuA1*	ABC-type Zn/Mn transporter, substrate-binding protein	TGTTGACATCCTTTTTCAATA	3.52	43.8
*cg2912*	*znuC1*	ABC-type Zn/Mn transporter, ATPase subunit	-	2.79	75.8
*cg2913*	*znuB1*	ABC-type Zn/Mn transporter, permease subunit	-	1.29	29.0

^1 ^Genes listed without 21-bp motif (-) belong to predicted operons.

^2 ^The gene expression in *C. glutamicum *JS2502 was compared with that of the wild-type strain ATCC 13032. Array, *m*-values obtained by DNA microarray hybridizations (intensity ratio); RT-PCR, values obtained by RT-PCR (relative expression).

^3 ^First gene of the putative *cg0042*-*cg0041*-*cg0040 *operon.

^4 ^First gene of the putative *cg2911*-*cg2912*-*cg2913 *operon.

The DNA microarray hybridization revealed 15 additional genes that were differentially expressed in the *zur *mutant *C. glutamicum *JS2502 (Table 2). As most of the corresponding *m*-values were close to the detection limit of the DNA microarray, expression of this gene set was furthermore examined by real-time RT-PCR. Using this more sensitive detection method, the expression of nine genes turned out to be significantly up-regulated in the *zur *mutant (Table 2). Among several coding regions of unknown function, this gene set includes *cg1447 *coding for a putative cobalt/zinc/cadmium efflux transporter and *cg3096 *(*ald*) encoding acetaldehyde dehydrogenase. In conjunction with the zinc-dependent alcohol dehydrogenase, the Ald protein is involved in the two-step utilization of ethanol as sole carbon and energy source by *C. glutamicum *[38]. As none of the genes is preceded by a candidate Zur-binding site, differential expression in *C. glutamicum *JS2502 is most likely a secondary effect of the *zur *gene deletion.

**Table 2:** Differentially expressed genes in the *zur *mutant *C. glutamicum *JS2502 detected by DNA microarray hybridization and lacking candidate Zur-binding sites.

CDS	Gene	Predicted function	Differential gene expression^1^	
			Array	RT-PCR^2^
*cg0045*	-	ABC-type transporter, permease subunit	- 1.12	n.s.
*cg0215*	*cspA*	cold-shock protein A	1.24	n.s.
*cg0793*	-	putative secreted protein	1.67	4.01
*cg0796*	*prpD1*	citrate dehydratase	1	6.68
*cg1109*	*porB*	anion-specific porin precursor	1.12	7.67
*cg1332*	-	putative secreted protein	1.42	2.57
*cg1447*	-	putative Co^2+^/Zn^2+^/Cd^2+ ^efflux transporter	3.16	25.8
*cg1670*	-	hypothetical protein	1.07	4.81
*cg2181*	-	ABC-type transporter, substrate-binding protein	1.1	4.91
*cg2261*	*amtB*	secondary ammonium transporter	- 1.01	n.s.
*cg2560*	*aceA*	isocitrate lyase	1.56	n.s.
*cg2925*	*ptsS*	phosphotransferase system component	- 1.35	n.s.
*cg3096*	*ald*	acetaldehyde dehydrogenase	1.69	103
*cg3138*	-	putative membrane protease subunit	3.14	n.s.
*cg3139*	-	hypothetical protein	2.03	n.s.
*cg3140*	*tagA1*	DNA-3-methyladenine glycolase I	1.88	n.s.
*cg3195*	-	putative flavin-containing monooxygenase	1.44	3.22

^1 ^Gene expression in *C. glutamicum *JS2502 was compared with that of the wild-type strain ATCC 13032. Array, *m*-values obtained by DNA microarray hybridizations (intensity ratio); RT-PCR, values obtained by RT-PCR (relative expression).

^2 ^Abbreviation: n.s., no significant differences detected.

### Verification of differential gene expression and promoter mapping

To support the conclusion that Zur is involved in transcriptional regulation of the potential target genes, control assays with a complemented *C. glutamicum zur *mutant were performed, thereby measuring the differential gene expression by RT-PCR. For this purpose, the *zur *gene was amplified by PCR and cloned into the *C. glutamicum *expression vector pEC-XK99E, resulting in plasmid pEC-XK99E_*zur *(Table 3). First, the differential expression of potential Zur target genes in *C. glutamicum *JS2502 was verified by real-time RT-PCR assays. As expected, the mRNA levels of all genes were clearly enhanced in the *zur *mutant when compared with the wild-type strain (Table 1), with the exception of the *adhA *gene (data not shown). Additional RT-PCR assays with the complemented strain *C. glutamicum *JS2502 [pEC-XK99E_*zur*] showed that the expression of potential target genes was indistinguishable from that of the wild-type strain ATCC 13032 carrying the empty cloning vector pEC-XK99E (data not shown). These results clearly demonstrated that the observed deregulation of gene expression can be attributed to the defined deletion of the *zur *gene in *C. glutamicum *JS2502.

**Figure 6:**
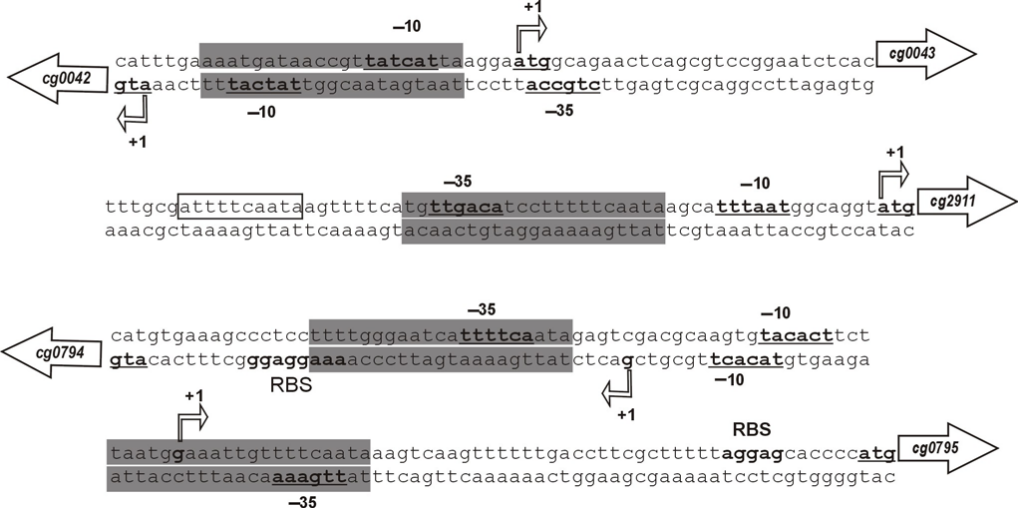
**Promoter organization of the Zur regulon members in *C. glutamicum *ATCC 13032**. A schematic presentation of relevant DNA regions from the *C. glutamicum *ATCC 13032 genome with detected promoters and candidate Zur-binding sites is presented. The 21-bp motifs are shown as grey boxes. A stretch of ten nucleotides (boxed), located upstream of the *cg2911 *promoter region, revealed similarity to the right half site of the 21-bp motif. The transcription start sites (+1) were mapped by 5' RACE-PCR and are marked in bold letters. Underlined nucleotides show the deduced - 10 and - 35 regions belonging to the corynebacterial promoters. Putative ribosome-binding sites (RBS) are indicated, start codons are underlined. The transcription start site and the - 10 region of *cg0043 *were deduced from bioinformatic predictions.

To elucidate whether the detected candidate Zur-binding motif is relevant for transcriptional regulation of the respective genes by Zur, the transcription start sites were determined by 5' RACE-PCR (Fig. 6). The mapped transcription sites were used to deduce thereof the respective promoter regions according to the corynebacterial consensus sequences for - 10 and - 35 regions [29]. The transcription start sites in front of *cg0042*, *cg0043 *and *cg2911 *were identical to the adenine residue of the respective ATG start codons, indicating the presence of so-called leaderless transcripts that were detected previously in *C. glutamicum *[29]. In all cases, the candidate Zur-binding motif overlaps the deduced core promoter regions (Fig. 6). Due to the short intergenic region (29 bp) between *cg0042 *and *cg0043*, a single candidate Zur-binding motif can be used to control the expression of the divergently oriented transcription units. The genetic organization of the *cg0794*-*cg0795 *intergenic region (118 bp) is more remarkable, as the motifs overlapping the - 35 region in front of *cg0794 *or *cg0795 *are both simultaneously located downstream of the - 10 region belonging to the other gene (Fig. 6). These locations of the candidate Zur-binding motifs are consistent with the positions of operators used by repressor proteins to exert negative transcriptional control of gene expression [39]. As the Zur binding sites always overlap either the - 35 region or the entire - 10/- 35 region of its target promoters, Zur binding can block the entry of the RNA polymerase and thereby repress the transcription of the target genes. On the other hand, the candidate Zur-binding motif detected in the *adhA *gene region is located 167 nucleotides upstream of the mapped transcription start site [37]. These experimental data therefore indicated that five transcription units (*cg0040*-*cg0041*-*cg0042*/*cg0043*, *cg2911*-*cg2912*-*cg2913*, *cg0794*/*cg0795*) are under negative transcriptional regulation by Zur in *C. glutamicum*.

### Verification of zinc-dependent expression of the putative *cg0042 *and *cg2911 *operons

**Figure 7:**
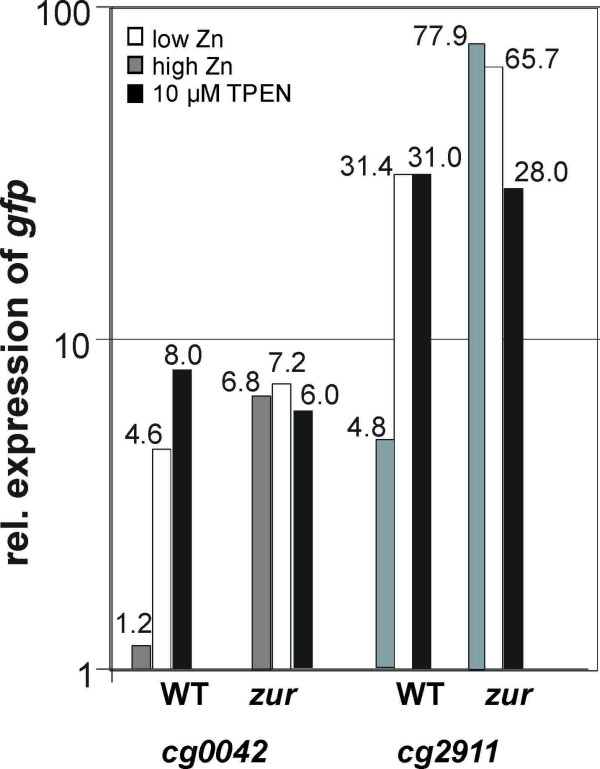
**Zinc-dependent activity of the *cg0042 *and *cg2911 *operon promoters**. The promoter activities of the Zur-regulated operons *cg0042 *and *cg2911 *was measured in the wild-type strain *C. glutamicum *ATCC 13032 (WT) and in the *zur *mutant *C. glutamicum *JS2502 (*zur*) under low, high and zinc-chelated (TPEN) conditions. The relative expression of the *gfp *reporter gene was determined by real-time RT-PCR. The values are means of four measurements. The relative expression was calculated by using a *C. glutamicum *control carrying the empty expression vector pEPR1.

As the putative operons *cg0042 *and *cg2911 *are apparently under negative control by the Zur protein in *C. glutamicum*, we investigated their zinc-dependent expression *in vivo *by using again the promoterless *gfp *reporter system. For this purpose, the mapped promoter regions were amplified by PCR and cloned into the promoter-probe vector pEPR1. The resulting plasmids pEPR1_prom_*cg0042 *and pEPR1_prom_*cg2911 *(Table 3) were transferred into the *C. glutamicum *ATCC 13032 wild-type strain and into the *zur *mutant *C. glutamicum *JS2502 to detect differential *gfp *expression by real-time RT-PCR, using high, low and chelated zinc conditions in the growth medium (Fig. 7). *C. glutamicum *ATCC 13032 carrying the empty cloning vector pEPR1 served as reference for calculating the differential gene expression. In the wild-type strain, the cloned promoters are apparently repressed under high-zinc condition and are derepressed under zinc-depletion, i.e. low-zinc condition and during growth in the presence of the chelator N,N,N',N'-tetrakis-(2-pyridylmethyl)-ethylenediamine (TPEN). A similar deregulation of gene expression was detected in the *zur *mutant *C. glutamicum *JS2502, irrespective of the presence or absence of zinc ions in the growth medium (Fig. 7). These *in vivo *data suggested that the lack of zinc-dependent regulation of gene expression is caused by the absence of the Zur protein in *C. glutamicum *JS2502. Furthermore, the data indicated that the Zur protein is sensing zinc ions and that it binds to operator sequences in the presence of zinc, thus acting as a repressor of the *cg0042 *and *cg2911 *operons.

### Verification of predicted Zur binding sites by *in vitro *DNA band shift assays

**Figure 8:**
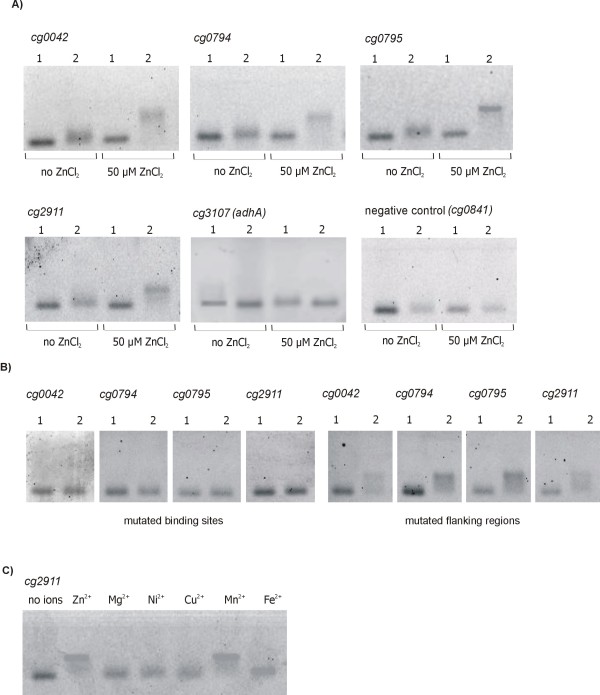
**Agarose gels of DNA band shift assays with purified Zur protein**. **(A)**, The DNA band shift assays with fluorescein-labeled 40-mers covering the candidate Zur-binding sites in the *cg0042*-*cg0043 *intergenic region and in front of *cg0794, cg0795*, *cg2911*, and *cg3107*. DNA band shift assays were performed with 40 pmol of streptavidin-tagged Zur protein incubated with 0.05 pmol of fluorescein-labeled, double-stranded 40-mer DNA fragments. The assays were performed in the absence of zinc ions and in the presence of 50 μM ZnCl_2_. Lanes 1: control assays without Zur protein; lanes 2: DNA band shift assays with added Zur protein. The negative control assay was performed with a 40-mer deduced from the upstream region of *cg0841. (B)*, DNA band shift assays with mutated versions of the 40-mers. Mutated versions were generated by introducing transitions into the candidate Zur-binding sites or into the genomic flanking regions. The EMSAs were carried out in the presence of 50 μM ZnCl_2_. **(C)**, DNA band shift assays with binding buffers containing varying metal ions. The EMSAs were performed in the presence of 50 μM ZnCl_2_, MgSO_4_, NiCl_2_, CuSO_4_, MnSO_4_, or FeSO_4 _with the 40-mer region representing *cg2911*.

To demonstrate experimentally the direct interaction of Zur with the candidate Zur-binding motifs detected in front of potential target genes, EMSAs were performed using fluorescein-labeled 40-mer oligonucleotides containing the 21-bp motif in the center of native genomic sequences (Fig. 8). For this purpose, the *C. glutamicum *Zur protein was tagged with streptavidin and purified by means of Strep-Tactin sepharose-packed columns (data not shown). Retardation of the respective double-stranded 40-mer DNA fragments was observed when the purified Zur protein and 50 μM ZnCl_2 _were added to the DNA band shift assays (Fig. 8A). In the absence of ZnCl_2 _no *in vitro *interaction of the purified Zur protein with the 40-mer DNA fragments was detected. A 40-mer sequence representing a regulatory gene region with a LexA binding site located in front of *cg0841 *[40] served as additional negative control. Likewise, Zur did not interact *in vitro *with the 21-bp motif located upstream of the *adhA *promoter region (Fig. 8A). Furthermore, mutated versions of the 21-bp motifs were generated by introducing transitions (Fig. 8B). In these cases, the purified Zur protein failed to shift the mutated DNA sequences. On the other hand, transitions introduced into the DNA segments flanking the 21-bp motifs did not affect the *in vitro *binding of Zur (Fig. 8B). To better define the role of metals in the ability of Zur to interact with its operators, EMSAs were performed in the presence of either 50 μM ZnCl_2_, MgSO_4_, NiCl_2_, CuSO_4_, MnSO_4_, or FeSO_4 _using exemplarily the 21-bp motif located upstream of *cg2911 *(Fig. 8C). These assays showed that the purified Zur protein was able to interact with this DNA fragment *in vitro *in the presence of either zinc or manganese ions. Similar observations were reported from DNA binding assays with Zn-dependent regulators from *M. tuberculosis *[31] and *B. subtilis *[41]. The four 21-bp motifs recognized by the purified Zur protein *in vitro *were used to delineate their consensus sequence in *C. glutamicum *ATCC 13032, which is highly similar to the FurB (Zur) consensus binding site from *M. tuberculosis *that was defined experimentally by DNase I footprint analysis [31] (data not shown). In summary, these results demonstrated the specific interaction of the Zur protein with the 21-bp operator motif in the presence of zinc, thereby negatively controlling the expression of nine genes belonging to the Zur regulon in *C. glutamicum *ATCC 13032.

## Discussion

### The *zur *gene encoding a zinc uptake regulator is conserved in genomes of actinobacteria

In the present study, we have examined the regulatory role of the *C. glutamicum *Zur protein (Cg2502) in the direct transcriptional control of gene expression. Zur was classified by protein domain pattern analysis as member of the Zur subgroup of the Fur protein family [4]. Fur proteins form a ubiquitous group of metal-responsive transcription regulators in many diverse bacterial lineages [14,42-44]. Comparative genomics revealed the presence of more than one *fur *homologue in most members of the taxonomic class *Actinobacteria *whose genome sequences have been completely determined, indicating that a gene duplication event predated the appearance of the last common ancestor of the actinobacteria [45]. A corresponding evolutionary model suggested that the resulting paralogues maintained the main biochemical properties of the ancestor regulator, but became specialized for coordinating different metal ions [45,46], including iron (Fur), manganese (Mur), nickel (Nur), and zinc (Zur) [14]. An apparent gene loss event occurred in the common ancestor of the corynebacteria, as *Corynebacterium *genomes do not contain the *furA *gene encoding a regulator for oxidative stress genes, but have the orthologous *furB *(*zur*) genes [45]. Accordingly, the *zur *gene product of *C. glutamicum *belongs to the small set of 24 transcription regulators that were detected in all hitherto sequenced corynebacterial genomes [5,19]. Moreover, synteny analyses revealed a conserved chromosomal region surrounding the *zur *gene in corynebacteria and other actinobacteria, including *Mycobacterium*, *Nocardia *and *Rhodococcus *species [45]. In these species, the *zur *gene is located downstream of another regulatory gene encoding a putative metal-sensing transcription regulator of the SmtB/ArsR protein family [10,45]. Both regulators might be involved in controlling the balanced expression of genes involved in zinc uptake and metabolism in some actinobacteria [26,47]. In *M. tuberculosis*, the *rv2358*-*furB *operon is (auto)regulated by Rv2358 [26] and functions as the regulatory interface between the control of zinc uptake and efflux [47]. At low zinc concentrations, Rv2358 negatively regulates expression of the *zitA *gene for a zinc efflux system [31] and the transcription of *furB*, thereby enabling the expression of FurB-regulated genes, including genes for zinc uptake systems [26]. At high zinc concentrations, Rv2358 does not bind to the operator site in front of the *rv2358*-*furB *operon and, as a consequence, zinc uptake is prevented by the regulatory action of FurB and an excess of zinc is pumped out of the cell. Since the genomic localization and the transcriptional organization of the *znr*-*zur *operon in *C. glutamicum *ATCC 13032 is similar to that of *M. tuberculosis *H37Rv, the regulatory role of Cg2500 (Znr) might be similar to that of Rv2358, i.e. both transcription regulators work together to optimally balance the zinc concentration in the *C. glutamicum *cell. To verify this conclusion, the target genes of Znr and its zinc-dependent interaction with the corresponding regulatory DNA sites have to be determined in *C. glutamicum *in future studies.

### The set of genes differentially expressed in the zur-mutant *C. glutamicum *JS2502 partially overlaps with the ethanol stimulon of *C. glutamicum*

The combination of genome-wide transcriptional profiling by DNA microarray hybridization and *in vitro *DNA band shift assays clearly demonstrated that the *C. glutamicum *Zur protein negatively controls the expression of five transcription units with genes that are involved in the zinc metabolism in this species. A comparison of the transcriptomes of the *zur*-deficient mutant *C. glutamicum *JS2502 and the wild-type strain *C. glutamicum *ATCC 13032 revealed 18 genes with increased expression in the *zur *mutant JS2502. This gene set, representing the cellular response to *zur*-deficiency in *C. glutamicum *JS2502, partially overlaps with a stimulon detected recently in *C. glutamicum *ATCC 13032 cells grown with ethanol as the sole carbon and energy source [38]. Growth of *C. glutamicum *ATCC 13032 on ethanol was characterized by enhanced expression levels of 36 genes when compared with acetate- and glucose-grown cultures. The set of differentially expressed genes detected in both genome-wide profiling studies include: (i) *cg0040 *to *cg0043 *and *cg2911 *to *cg2913 *encoding putative ABC-type uptake systems for zinc ions, (ii) *cg3096 *encoding acetaldehyde dehydrogenase and (iii) *cg3195 *encoding a putative flavoprotein. On the other hand, an enhanced expression of the Zur regulon members *cg0794 *and *cg0795 *was not deteced during growth of *C. glutamicum *on ethanol. The genes for the putative zinc uptake systems showed the largest increase of mRNA levels in ethanol-grown cells of *C. glutamicum*, which was explained by the higher demand of zinc due to its incorporation into the zinc-dependent alcohol dehydrogenase (AdhA) of *C. glutamicum *[38]. A candidate Zur-binding site was detected by cross-genome comparisons in the upstream region of *adhA *(*cg3107*), but the purified Zur protein did not bind to a corresponding 40-mer DNA sequence *in vitro*. In addition, the *adhA *gene was not detected as differentially expressed in the *zur*-deficient mutant *C. glutamicum *JS2502. Therefore, our results did not provide any evidence that the candidate Zur-binding site is involved in transcriptional regulation of *adhA *gene expression. The integration of the detected regulatory interactions into the database CoryneRegNet [48,49] revealed that the Zur regulon forms a separate module in the transcriptional gene regulatory network model of *C. glutamicum *and is thus not linked to the currently known network supercluster [17]. Whether an additional carbon source-dependent control of the Zur regulon by any kind of coregulation or hierarchical interaction is established in *C. glutamicum *remains to be elucidated.

### Physiological function of genes belonging to the Zur regulon of *C. glutamicum*

Since the metal ions sensed by members of the Fur protein family are considered, on the one hand, fundamental for bacterial growth and, on the other hand, toxic at elevated levels, a strict balance between metal ion uptake and efflux is essential for homeostasis [14]. The target genes of the *C. glutamicum *Zur protein detected in this study include two putative ABC-type transport systems (Cg0041-Cg0043 and Cg2911-Cg2913), a putative secreted protein (Cg0040), a putative oxidoreductase (Cg0795), and a putative P-loop GTPase of the COG0523 family (Cg0794) that may specifically bind Zn^2+ ^ions [50]. We also showed that Zur binds to the predicted operator sequences located in the mapped promoter regions of the respective genes, which are therefore under direct negative transcriptional control. The deduced genetic organization of the *cg0794*-*cg0795 *intergenic region and the common transcriptional control of both genes *via *two Zur operator sites suggests a functional link between the respective proteins. Since some experimentally characterized members of the COG0523 protein family of P-loop GTPases are so-called metallochaperones, such as HypB from *Methanocaldococcus jannaschii *[51] and UreG from *Helicobacter pylori *[52], the *C. glutamicum *P-loop GTPase Cg0794 may also function (eventually in conjuction with the oxidoreductase Cg0795) as a zinc-specific metallochaperone/insertase to enable the *in vivo *assembly of zinc-containing proteins under environmental conditions of zinc deficiency. Furthermore, Cg0794 is similar to YciC, an abundant protein from *B. subtilis *postulated to function as a metallochaperone [35]. Expression of *yciC *in *B. subtilis *occurs in a zinc-dependent manner that is exerted by the *B. subtilis *Zur orthologue [49]. YciC-like proteins are often members of the Zur regulons in proteobacteria and firmicutes and may be involved in the specific binding and allocation of Zn^2+ ^ions [53]. The Cg2911 (ZnuA1) and Cg0041 (ZnuA2) proteins of *C. glutamicum *belong to the TroA superfamily of metal-binding proteins that are predicted to function as initial receptors in ABC-type transport systems of metal ions [21], supporting the view that both systems are involved in transport of divalent metal ions, such as Zn^2+^. The transcriptional regulation of genes encoding zinc uptake systems by Zur proteins seems to be common in actinobacteria, as the *zur *gene is located adjacent to *znu *operons in *Arthrobacter*, *Leifsonia*, *Acidothermus*, *Nocardioides*, *Streptomyces*, *Thermobifida*, and *Rubrobacter *species [45]. Likewise, genes encoding zinc ABC-type transport systems are under transcriptional control by Zur in *Streptococcus suis *[54], *Xanthomonas campestris *[55] and *Yersinia pestis *[56].

The transcriptional regulation of *znu *operons was characterized during genome-wide analyses of zinc-responsive regulators in *M. tuberculosis *H37Rv and *Streptomyces coelicolor *A3(2) [31-33]. The genes regulated by Zur_Mtub _encode three putative metal transporters, a group of ribosomal proteins and proteins belonging to the early secretory antigen target 6 (ESAT-6) cluster and the ESAT-6/CFP-10 (culture filtrate protein 10) family [52]. Likewise, Zur_Scoe _controls the expression of *znuACB*, located upstream of *zur *and encoding a zinc uptake transporter, and of genes for paralogous forms of ribosomal proteins that are devoid of zinc-binding motifs and can therefore replace, during zinc deficiency, their zinc-binding counterparts that can serve as zinc storage forms [32,33]. Three DNA binding sites of Zur_Scoe _were determined by DNase I footprinting analysis, revealing the 7-1-7 inverted repeat TGAAAATGATTTTCA as consensus sequence of potential operator sites [33]. This consensus sequence is similar to the central region of the 10-1-10 inverted repeat (candidate Zur-binding site) detected in the *C. glutamicum *genome in the present study. Likewise, DNA protection assays were used to identify Zur binding sites in the *M. tuberculosis *genome sequence [31]. The deduced 10-1-10 inverted repeat is also similar to the consensus sequence of Zur binding sites detected in the genome of *C. glutamicum*. Accordingly, the Zur binding sites in actinobacteria are apparently represented by a conserved 21-bp palindromic sequence with a 1-bp non-palindromic center, as shown by the Zur-binding motif sequence logo (Fig. 4B).

## Conclusions

The combination of cross-genome comparison of shared regulatory sites and whole-genome expression profiling with DNA microarrays allowed us to deduce the Zur regulon of *C. glutamicum *ATCC 13032. It consists of five transcription units covering nine genes and encoding the components of two potential ZnuACB zinc transporters, a putative secreted protein, a putative oxidoreductase, and a putative P-loop GTPase of the COG0523 protein family. *In vivo *expression studies and *in vitro *DNA band shift assays demonstrated that Zur directly represses the expression of its target genes in a zinc-dependent manner. Accordingly, the Zur (Cg2502) protein is the key transcription regulator for genes involved in zinc homeostasis in *C. glutamicum*.

## Methods

### Bacterial strains, plasmids and growth conditions

Bacterial strains and plasmids used and constructed in this study are listed in Table 3. *E. coli *DH5α MCR was grown at 37°C in Luria-Bertani medium [57] and used for standard cloning procedures as well as for heterologous expression of the *C. glutamicum *Zur protein. The induction of gene expression on the pASK-IBA5+ plasmid was carried out in *E. coli *DH5αMCR using 200 ng ml^-1 ^tetracycline. The wild-type strain *C. glutamicum *ATCC 13032 and the *zur *mutant *C. glutamicum *JS2502 were routinely grown at 30°C in CGXII minimal medium containing 30 μg l^-1 ^protocatechuic acid and 420 μg l^-1 ^thiamine [58]. Antibiotics for plasmid selection were kanamycin (50 μg ml^-1 ^for *E. coli *and 25 μg ml^-1 ^for *C. glutamicum*) and ampicillin (200 μg ml^-1 ^for *E. coli*). The growth of shake-flask cultures was monitored by measuring the optical density at 600 nm with an Eppendorf *Bio*Photometer.

**Table 3:** Bacterial strains and plasmids used in this study.

Strain or plasmid	Relevant characteristics	Source or reference
*C. glutamicum *ATCC 13032	wild-type strain	ATCC
*C. glutamicum *JS2502	ATCC 13032 with defined deletion in *zur*	This study
*E. coli *DH5αMCR	*E. coli *strain used for standard cloning procedures	[73]
*E. coli *TOP10	*E. coli *strain used for cloning of RACE-PCR products	Invitrogen
pCR2.1-TOPO	*lacZα*, Ap^r^; *E. coli *cloning vector	Invitrogen
pK18*mobsacB*	*sacB*, Km^r^; *E. coli *vector for allelic exchange	[74]
pK18*mobsacB*_Δ*zur*	*sacB*, Km^r^; pK18*mobsacB *carrying a modified *zur *gene with internal deletion	This study
pASK-IBA5+	*P*_Tet_, strep-tag, Ap^r^; *E. coli *expression vector	IBA Tagnologies
pASK-IBA5+_*cg2502*	pASK-IBA5+ carrying the *C. glutamicum zur *gene	This study
pEPR1	*gfpuv*_*PL*_, Km^r^; promoter-probe vector	[28]
pEPR1_prom_*cg2500*	*gfpuv*_PL_, Km^r^; pEPR1 carrying the *znr *upstream region	This study
pEPR1_prom_*cg2502*	*gfpuv*_PL_, Km^r^; pEPR1 carrying the *zur *upstream region	This study
pEPR1_prom_*cg0042*	*gfpuv*_PL_, Km^r^; pEPR1 carrying the *cg0042 *upstream region	This study
pEPR1_prom_*cg2911*	*gfpuv*_PL_, Km^r^; pEPR1 carrying the *cg2911 *upstream region	This study
pEC-XK99E	P_*trc*_, *lacI*, Km^r^; *C. glutamicum *expression vector	[75]
pEC-XK99E_*zur*	P_*trc*_, *lacI*, Km^r^; pEC-XK99E vector carrying the *zur *gene for complementation	This study

### DNA preparation and PCR techniques

The preparation of plasmid DNA from *E. coli *cells was performed by the alkaline lysis technique using the QIAprep Spin Miniprep Kit (Qiagen). The protocol was modified for *C. glutamicum *cells by using 20 mg ml^-1 ^lysozyme in resuspension buffer P1 and by incubating the assay at 37°C for 3 h. Chromosomal *C. glutamicum *DNA was isolated as described previously [59]. DNA restriction, analysis by agarose gel electrophoresis and DNA ligation were performed according to standard procedures [57]. The transformation of plasmid DNA was carried out by electroporation using electrocompetent *E. coli *and *C. glutamicum *cells [60,61]. The DNA amplification by PCR was performed with a PTC-100 thermocycler (MJ Research) and BIOTAQ DNA polymerase (Bioline) or Phusion Hot Start High-Fidelity DNA polymerase (Finnzymes). The PCR products were purified with the PCR Purification Spin Kit (Qiagen). All Oligonucleotides used in this study were purchased from Operon Biotechnologies (see additional file 2).

### Construction of a defined zur deletion in *C. glutamicum*

The gene SOEing procedure [62] was applied to establish a defined deletion of 195 nucleotides in the *zur *coding region. The PCR primers used for gene SOEing were *cg2502*del1 to *cg2502*del4 (see additional file 2). The resulting pK18*mobsacB *derivative, pK18*mobsacB*_Δ*zur *(Table 3), was applied to perform an allelic exchange by homologous recombination in the chromosome of *C. glutamicum *ATCC 13032 [63], resulting in the mutant strain *C. glutamicum *JS2502. To complement the *zur *mutant phenotype, a DNA fragment covering the complete coding region of *zur *was amplified by PCR with the primer pair *cg2502_*compl1 and *cg2502_*compl2 (see additional file 2), digested with EcoRI and BamHI, and cloned in *E. coli *into the corresponding sites of shuttle expression vector pEC-XK99E (Table 3).

### Testing *in vivo *promoter activity

The upstream region of the *zur *(*cg2500*) gene and the *znr*-*zur *intergenic region were amplified from chromosomal *C. glutamicum *DNA by PCR with the primer pairs *cg2500*_GFP1-*cg2500*_GFP2 and *cg2502*_GFP1-*cg2502*_GFP2, respectively (see additional file 2). The PCR products were digested with appropriate enzymes and cloned into compatibel sites of the promoter-probe vector pEPR1 [28] that contains the promoterless *gfp *reporter gene coding for the green fluorescent protein. The reporter gene of pEPR1 will be expressed only if the DNA fragment cloned in front of *gfp *contains an active promoter [28]. The expression of the *gfp *gene in *E. coli *DH5αMCR and *C. glutamicum *ATCC 13032 was detected by fluorescence microscopy with an Axiophot microscope (Zeiss) at a 400-fold magnification. All digital GFP pictures were taken with an exposure time of four seconds.

### Measurement of *in vivo *promoter activity for *cg0042 *and *cg2911*

To detect a zinc-dependent expression of the *cg0042 *and *cg2911 *operons, approx. 200 bp segments covering the respective core promoter regions were amplified from chromosomal *C. glutamicum *DNA by PCR with the primer pairs *cg0042*_GFP1-*cg0042*_GFP2 and *cg2911*_GFP1-*cg2911*_GFP2, respectively (see additional file 2). The PCR products were cloned in *E. coli *DH5αMCR into the promoter-probe vector pEPR1, providing a promoterless *gfp *reporter gene for subsequent measurements [28]. The plasmids were transformed into *C. glutamicum *ATCC 13032 and the *zur *mutant *C. glutamicum *JS2502 by electroporation. The resulting strains were grown in CGXII minimal medium containing 1 mg l^-1 ^ZnSO_4 _(high Zn condition) and in CGXII without additional ZnSO_4 _(low Zn condition). Additionally, the cells were exposed to 10 μM of the chelator N,N,N',N'-tetrakis-(2-pyridylmethyl)-ethylenediamine (TPEN) for 3 h in CGXII minimal medium (Zn-chelated condition). Expression of the *gfp *reporter gene was measured by real-time RT-PCR using the primers LCPrimer1_*gfp *and LCPrimer2_*gfp *(see additional file 2).

### RNA techniques and DNA microarray hybridizations

The isolation and purification of total RNA from *C. glutamicum *cells was carried out as described previously [64]. The transcript levels of genes were measured by real-time reverse transcription PCR (RT-PCR) with the LightCycler instrument (Roche Applied Scince), using the SensiMix One-Step Kit (Quantace). The differences in gene expression between *C. glutamicum *JS2502 and the wild-type strain ATCC 13032 were determined by comparing the crossing points of two biological samples, each measured with two technical replicates. The measured crossing point (CP) is the cycle at which PCR amplification begins its exponential phase and is considered the point that is most reliably proportional to the initial RNA concentration (Roche Applied Science). The amounts of the mRNAs of the genes were normalized on total RNA, and the relative change in transcription rate was determined as 2-Δ^CP^, with ΔCP equal to the difference of the measured crossing points for the test and the control condition. The crossing points were calculated by the LightCycler software (Roche Applied Science). The quality of the measurement was ensured by melting curve analysis.

Transcription start sites were determined by using the 5'/3' RACE Kit second generation according to the manufacturer's instructions (Roche Applied Science). Starting with 1 μg of total *C. glutamicum *RNA, this approach enables the transcription of gene specific mRNA sequences into first-strand cDNA with the cDNA synthesis primer SP1 (see additional file 2). This initial cDNA synthesis was followed by a further amplification with nested PCR using the gene specific primer SP2 (see additional file 2). All PCR procedures were performed according to the recommendations of the manufacturer (Roche Applied Science) with a PTC-100 thermocycler (MJ Research). The PCR products were cloned into the pCR2.1-TOPO vector using the TOPO TA Cloning Kit (Invitrogen), and the resulting plasmids were transferred into chemically competent *E. coli *TOP10 cells. The cloned RACE-PCR products were finally sequenced to determine the 5' end of the mRNA (IIT Biotech).

For global transcription profiling, hybridization of whole-genome DNA microarrays was performed with total RNA probes isolated from two independently grown *C. glutamicum *cultures. The respective cDNA samples were labeled with Cy3/Cy5 in one experiment and with Cy5/Cy3 in the other one (label swapping). Since each *C. glutamicum *DNA microarray contains four spots per gene, a maximum of eight spots per gene provided data for calculating differential gene expression. To minimize the number of false-positive signals, hybridization data were stringently filtered to obtain genes with at least six statistically significant values out of the eight technical replicates, applying an error probability of less than 5% for the *t*-test [64]. The data normalization was carried out with the LOWESS function, and *t*-test statistics were calculated with the EMMA2 software package [65]. The microarray hybridization data were deposited in the CoryneRegNet database with identifier "delta_zur" and can be downloaded for further analysis by using SOAP-based web services [66].

### Overexpression and purification of the *C. glutamicum *Zur protein

To fuse the *C. glutamicum *Zur protein with an amino-terminal streptavidin (strep)-tag, the coding region of the *zur *gene was amplified by PCR with the primer pair *cg2502*_fwd_5Strep and *cg2502*_rev_5Strep (see additional file 2), which were created by using the IBA Primer D'Signer1.1 software (IBA BioTAGnology). The resulting PCR product was digested with BsaI and cloned into pASK-IBA5+ to give plasmid pASK-IBA5+_*cg2502 *(Table 3) that was transferred to *E. coli *DH5α MCR. Cell culturing, overexpression of the recombinant Zur protein and purification with Strep-Tactin sepharose-packed columns were carried out according to the manufacturer's instructions. The RiboLyser instrument was used for cell disruption, with a speed rate of 6.5 for two time intervals of 30 s and ice-cooling of 1 min in-between. The concentration of the eluated protein was determined with the Bio-Rad protein assay kit (Bio-Rad Laboratories), and the eluate was analyzed by SDS-PAGE. To verify the purification of the Zur protein, an in-gel digestion with modified trypsin (Promega) was carried out. A peptide mass fingerprint of the purified protein was determined by matrix-assisted laser desorption/ionization time-of-flight (MALDI-TOF) mass spectrometry, applying an Ultraflex mass spectrometer (Bruker Daltonics) and the MASCOT software.

### DNA band shift assays with streptavidin-tagged Zur protein

Purified Zur protein was used in electrophoretic mobility shift assays (EMSAs) to determine its ability to interact with *in silico *predicted operators in dependence on zinc. EMSAs were performed using fluorescein-labeled 40-mer oligonucleotides that were annealed with complementary oligonucleotides to double-stranded DNA fragments by heating for 5 min at 94°C and cooling on ice for 15 min. The binding assays were performed in a final volume of 20 μl, containing 0.05 pmol of the double-stranded 40-mer, 40 pmol of strep-tagged Zur protein, 0.06 μg herring sperm DNA, and binding buffer (20 mM Tris-HCl, 50 mM KCl, 1 mM DTT, 50 μg ml^-1 ^bovine serum albumin, 5% glycerol; pH 8.0). EDTA was added to the binding reaction to a final concentration of 400 μM [31]. Ions (ZnCl_2_, MgSO_4_, NiCl_2_, CuSO_4_, MnSO_4_, or FeSO_4_) were added to EMSAs in a concentration of 50 μM. The assays were incubated at 30°C for 30 min and separated in 2% agarose gels prepared in gel buffer (40 mM Tris-HCl, 10 mM sodium acetate, 1 mM EDTA; pH 7.8). A voltage of 70 V was applied for 1 h. The agarose gels were scanned with a Typhoon 8600 Variable Mode Imager (Amersham Biosciences Europe).

### Bioinformatic methods and comparative genomic analysis of Zur regulons

The complete genomes of actinobacteria were downloaded from GenBank [67]. The *Actinobacteria*-specific training set for the identification of the Zur-binding motif was composed of the candidate zinc transporter genes *znuABC*. The DNA motif search profiles (a positional-weight matrix) were constructed using the SignalX program. Analyzed genomes were scanned with the constructed Zur-binding motif profile using the Genome Explorer software [68], and the identified genes with candidate Zur-binding sites were analyzed by the consistency check comparative procedure as previously described [6]. Positional nucleotide weights in the recognition profile and *Z*-scores of candidate sites were calculated as the sum of the respective positional nucleotide weights [69]. The threshold for the site search was defined as the lowest score observed in the training set (*Z*-score = 4.8). The sequence logo for the consensus Zur-binding motif in *Actinobacteria *was constructed using WebLogo 2.0 [70]. The phylogenetic trees were constructed by the maximum likelihood method implemented in the PROML program of the PHYLIP package [71] using multiple sequence alignments of protein sequences produced by the Clustal W2 program [72]. The deduced regulatory interactions were stored in the CoryneRegNet database [48].

## Authors' contributions

JS performed the experimental work and drafted the manuscript. NJ participated in experimental design and data evaluation. DAR performed the genome-wide detection of Zur regulons in actinobacteria. AT participated in data evaluation and supervision. All authors read and approved the final version of the manuscript.

## Supplementary Material

Additional file 1 **Candidate Zur-binding sites in the genomes of actinobacteria**. The Excel file contains a list of detected Zur binding sites and candidate Zur-regulated genes. Click here for file

Additional file 2 **Oligonucleotides used in this study**. The PDF contains a list of all oligonucleotides used in the present work. Click here for file
