# Supplementary material for: The Zur regulon of Corynebacterium glutamicum ATCC 13032
Source: BMC Genomics. 2010 Jan 7;11:12. doi: 10.1186/1471-2164-11-12 (PMC2823685; doi:10.1186/1471-2164-11-12)
Supplement: Additional file 2 — Oligonucleotides used in this study. The PDF contains a list of all oligonucleotides used in the present work. [file 1471-2164-11-12-S2.PDF]

Additional file 2: Oligonucleotides used in this study.

**Primers for gene SOEing**			
*cg2502*del1	GATCTAGAATTCACCACTCGTCCTCGACAT		
*cg2502*del2	TTCAGCCTCGTGACTACT-GGTTCGGTAGACGGTTGT		
*cg2502*del3	AGTAGTCACGAGGCTGAA		
*cg2502*del4	GATCTAGGATCCGTTGTCGATGGCGTTGA		
**Primers for protein tagging**			
*cg2502*_fwd_5Strep	ATGGTAGGTCTCAGCGCCGTGGGTATCAATCGCATCAGCC		
*cg2502*_rev_5Strep	ATGGTAGGTCTCATATCACGTAACTTTTTCCTTACAATCAGCG		
**Primers for promoter activity tests**			
*cg2502*_GFP1	GCGTCTCACTAGTATTCACTTGCCGAACCA		
*cg2502*_GFP2	GCGTCTCAGATCCGTTGATCCTCCTCAATG		
*cg2500*_GFP1	GCGTCTCACTAGATGTTGCTTCCGCTGACC		
*cg2500*_GFP2	GCGTCTCAGATCTCCGTCCTTAGGCTAGGT		
*cg0042*_GFP1	GCGTCTCACTAGCAAGCCGTGCGACATAGC		
*cg0042*_GFP2	GCGTCTCAGATCGAGCGCGATGTGATTGCC		
*cg2911*_GFP1	GCGTCTCACTAGGTTCTGCGGAGAGTTGCT		
*cg2911*_GFP2	GCGTCTCAGATCAGCAGCCAGCTAGTACCA		
**Primers for complementation**			
*cg2502*_compl1	GATCTAGAATTCGTGGGTATCAATCGCATC		
*cg2502*_compl2	GATCTAGGATCCGTCGAGCACTCACATGTT		
**Oligonucleotides for RACE-PCR**			
*cg0042*_SP1	CACTCCGGCTGACATGAT		
*cg0042*_SP2	TGCGCCGAACATGAGATT		
*cg0042*_SP3	TAGTGATGCCGTGGCTAC		
*cg0794*_SP1	CGGCATTGGTTCGGAGAT		
*cg0794*_SP2	CGGAATCCACGAGGTCTT		
*cg0794*_SP3	ATGCATCCGTTGGTCAGT		
*cg0795*_SP1	GCGTCGTCAAGCGTTATG		
*cg0795*_SP2	AAGCGTTATGGCGAAGGT		
*cg0795*_SP3	TGGTGACGCCATAGGAAC		
*cg2911*_SP1	CCGTAGAGCCAGGAATCA		
*cg2911*_SP2	CGCCACCGACGATAATGA		
*cg2911*_SP3	CGGAAGGCTCGAAGGAAT		
**Oligonucleotides for RT-PCR**			
LCPrimer1_*cg0041*	GATCTCATCGTGGCCAAT	LCPrimer1_*cg1447*	TTCAGCAGCGCAGTTATG
LCPrimer2_*cg0041*	CGGTGACTTCCTCATCAA	LCPrimer2_*cg1447*	GTAGGTCGCACGAGAAGT
LCPrimer1_*cg0042*	TAGCCACGGCATCACTAT	LCPrimer1_*cg3138*	GGAACACTTGCGGTTACG
LCPrimer2_*cg0042*	GTTGCAATGGCGATGATG	LCPrimer2_*cg3138*	TACCTGCCACACGATGAT
LCPrimer1_*cg0043*	CATGCACATACGGCAATC	LCPrimer1_*cg3139*	CCTTGATCCGGCAGTCTA

LCPrimer2_*cg0043*	CCACGATGTTGCAATCTG
LCPrimer1_*cg0794*	CCGCATCTACATCACCAA
LCPrimer2_*cg0794*	CTCCTTATGCCGTAGTCA
LCPrimer1_*cg0795*	CACCAAGCGTTGATGTTC
LCPrimer2_*cg0795*	CCATCTGACCACCAAGTT
LCPrimer1_*cg2911*	CTTCCGCTACCGATATGG
LCPrimer2_*cg2911*	CTGCGAACTCTTCAGCTA
LCPrimer1_*cg1912*	TGAATTGAAGGCCGAAGG
LCPrimer2_*cg2912*	AACCACCACGATTCTGTC
LCPrimer1_*cg2913*	GTCCGTGCTGTTCATTCA
LCPrimer2_*cg2913*	GATCAGCAATGCCATGAC
LCPrimer1_*cg3107*	CCAGGCATTGGTGAAgGGT
LCPrimer2_*cg3107*	CTGGCCGAAGGATCCATT
LCPrimer1_*cg3195*	AAGGATACTGGCGAGACC
LCPrimer2_*cg3195*	CCGTGCACTTGAAGTCTG
LCPrimer1_*cg1332*	TGGCGATGCCTATAGCTT
LCPrimer2_*cg1332*	ATTGCGGAATCGAGGACA
LCPrimer1_*cg0215*	CTCCGCAGATCTCTTCGT
LCPrimer2_*cg0215*	TGGCTGGTTTTCCTCAAG
LCPrimer1_*cg0045*	CGTGCTGTATGCGAGTTC
LCPrimer2_*cg0045*	TTGGTGACCGCCAGATTA
LCPrimer1_*gfp*	GACTTCAGCACGCGTCTT
LCPrimer2_*gfp*	TGGCCAACACTTGTCACT
LCPrimer2_*cg3139*	CATTAGTGCTGCGAGCTT
LCPrimer1_*cg3140*	CCGATTCGCGATGAACAG
LCPrimer2_*cg3140*	CGCCTTAGACAGTGCAAC
LCPrimer1_*cg3096*	CCGAAGGCTCGATCGTTA
LCPrimer2_*cg3096*	TAGCGGAAGTGGTCGATT
LCPrimer1_*cg0793*	GCACTGTCGAGCAACATT
LCPrimer2_*cg0793*	GCGCGTGTCTAGCATATT
LCPrimer1_*cg2560*	GCAGACCAGGTAGCTGAT
LCPrimer2_*cg2560*	TTGTTGATGCGACGAACG
LCPrimer1_*cg1109*	CATCACAGTGGCAGCATT
LCPrimer2_*cg1109*	TTGGAGGACAGCTCAGAA
LCPrimer1_*cg2181*	TCGTCGACAGCATCTTCT
LCPrimer2_*cg2181*	CTCGACGCCTTCTTCGTA
LCPrimer1_*cg1670*	CAACGAGCCTACGAACAA
LCPrimer2_*cg1670*	CCGAGCTAATTCCACCAT
LCPrimer1_*cg0796*	GTGGCCGTACGTGAATTG
LCPrimer2_*cg0796*	GATCGCCTGGTAGATGGT
LCPrimer1_*cg2925*	AGGCGATGTGGATCATGT
LCPrimer2_*cg2925*	TAGCCGTTCACCAAGCTC
LCPrimer1_*cg2261*	CGGCAATTCAGCTTGGAT
LCPrimer2_*cg2261*	GGAGATAACGGCGAAGGT
**DNA fragments for EMSAs**	
shift1_*cg0042*	TGCCATTCCTTAATGATAACGGTTATCATTTTCAAATGAA-Fluorescein
shift2_*cg0042*	TTCATTTGAAAATGATAACCGTTATCATTAAGGAATGGCA
shift1_*cg0795*	GTACACTTCTTAATGGAAATTGTTTTCAATAAAGTCAAGT-Fluorescein
shift2_*cg0795*	ACTTGACTTTATTGAAAACAATTTCCATTAAGAAGTGTAC
shift1_*cg2911*	TAAGTTTTCATGTTGACATCCTTTTTCAATAAGCATTTAA-Fluorescein
shift2_*cg2911*	TTAAATGCTTATTGAAAAAGGATGTCAACATGAAAACTTA
shift1_*cg0794*	GTCGACTCTATTGAAAATGATTCCCAAAAGGAGGGCTTTC-Fluorescein
shift2_*cg0794*	GAAAGCCCTCCTTTTGGGAATCATTTTCAATAGAGTCGAC
shift1_*cg3107*	TTgAAAAgTTAATTgAAAAACATTTCCATTAGGGGGTGAT-Fluorescein
shift2_*cg3107*	ATCACCCCCTAATGGAAATGTTTTTCAATTAACTTTTCAA
**DNA fragments with mutated motifs**	
*cg0042*_MotifMut1	TGCCATTCCTTGACGGTGATGATCACCGTCTTCAAATGAA-Fluorescein
*cg0042*_MotifMut2	TTCATTTGAAGACGGTGATCATCACCGTCAAGGAATGGCA
*cg0794*_MotifMut1	GTCGACTCCACTAAGAGTAACTTCTAGAGGGAGGGCTTTC-Fluorescein
*cg0794*_MotifMut2	GAAAGCCCTCCCTCTAGAAGTTACTCTTAGTGGAGTCGAC
*cg0795*_MotifMut1	GTACACTTCTCAGTAGGAGTCGATCTTAGTGAAGTCAAGT-Fluorescein

*cg0795*_MotifMut2	ACTTGACTTCACTAAGATCGACTCCTACTGAGAAGTGTAC
*cg2911*_MotifMut1	TAAGTTTTCACGCTAATACCTTCTCTTAGTGAGCATTTAA-Fluorescein
*cg2911*_MotifMut2	TTAAATGCTCACTAAGAGAAGGTATTAGCGTGAAAACTTA
**DNA fragments with mutated flanking regions**	
*cg0042*_Mut1	TACTACTTCCTAATGATAACGGTTATCATTTCCGAGTAAG-Fluorescein
*cg0042*_Mut2	CTTACTCGGAAATGATAACCGTTATCATTAGGAAGTAGTA
*cg0794*_Mut1	ATTGGCCCTATTGAAAATGATTCCCAAAAAGGGAGTTCTT-Fluorescein
*cg0794*_Mut2	AAGAACTCCCTTTTTGGGAATCATTTTCAATAGGGCCAAT
*cg0795*_Mut1	ATGCGCCTTTTAATGGAAATTGTTTTCAATAGAATTAGGC-Fluorescein
*cg0795*_Mut2	GCCTAATTCTATTGAAAACAATTTCCATTAAAAGGCGCAT
*cg2911*_Mut1	CAGGCTCTTATGTTGACATCCTTTTTCAATAGGTACTCAG-Fluorescein
*cg2911*_Mut2	CTGAGTACCTATTGAAAAAGGATGTCAACATAAGAGCCTG
